# Co-infection with *Mycobacterium tuberculosis* and *Nocardia farcinica* in a COPD patient: a case report

**DOI:** 10.1186/s12890-023-02434-3

**Published:** 2023-04-22

**Authors:** Yingzhu Chen, Wei Hu

**Affiliations:** 1 Department of Laboratory Medicine, Traditional Chinese Medicine Hospital of Dianjiang County, Chongqing, Chongqing, 408300 China; 2 Department of Renal Medicine, People’s Hospital of Dianjiang County, Chongqing, Chongqing, 408300 China

**Keywords:** Nocardia, Tuberculosis, Co-infection, COPD, Diagnosis

## Abstract

**Background:**

Chronic obstructive pulmonary disease (COPD) is a common respiratory disease characterized by persistent airflow limitation. Infection with either *Mycobacterium tuberculosis* or *Nocardia* in COPD patients has been reported. However, co-infection with *Mycobacterium tuberculosis* and *Nocardia* is rare. Herein, we described such a patient with COPD in a primary hospital, and the diagnosis process.

**Case presentation:**

A 79-year-old female farmer with COPD was consecutively admitted to two hospitals with chief complaints of worsening cough, sputum and gasping since January10, 2022. Microbiological examination was not performed at the first hospital due to unknown reasons, and empirical antibiotic treatment was not effective. The patient was subsequently referred to our hospital. After screening the source of infection and the pathogen, she was diagnosed with tuberculosis. However, the patient did not benefit from antituberculosis treatment, with no remission of respiratory tract symptoms. Cerebrospinal fluid and bronchoalveolar lavage fluid specimens were subsequently sent for microbiological examination. The results indicated *Mycobacterium tuberculosis* and *Nocardia.spp*. After four days of bacterial culture, *Nocardia.spp* grew on medium, and *Nocardia.farcinica* was identified by the MALDI-TOF MS system and 16 s RNA. The patient was prescribed trimethoprim sulfamethoxazole (TMP/SMX) in combination with anti-tuberculosis drugs to treat the co-infection. She showed gradual improvement and was discharged from the hospital on February 19, 2022. However, the follow-up results were unclear.

**Conclusions:**

Co-infection with *Nocardia* and *Mycobacterium tuberculosis* should be considered in COPD patients. Repeated microbiological and microscopy examinations are essential in primary hospitals.

## Background

Chronic obstructive pulmonary disease (COPD) is a common respiratory disease characterized by persistent airflow limitation [1]. Viruses and bacteria are the predominant pathogens causing respiratory tract infections in COPD patients. However, due to structural changes in the lungs and immunological changes within the respiratory system, these patients are also susceptible to *Mycobacterium tuberculosis* and relatively rare pathogens [2]. China has a high burden of tuberculosis and a lack of extensive research on nocardiosis. However, the common susceptibility factors for *Mycobacterium tuberculosis* and *Nocadia.spp* are underlying pulmonary diseases. A COPD patient has a three-fold higher risk of tuberculosis [3]. COPD is a confirmed to be a risk factor for pulmonary Nocardiosis (PN) [4, 5]. However, concurrent infection with these two pathogens is rare in patients with COPD. The rarity of co-infections leads to the possibility that the discovery of one pathogen may lower suspicion of the presence of other pathogens [6]. When patients with COPD are co-infected with these two pathogens, rapid and accurate identification of the two pathogens is crucial for accurate anti-infection therapy. This case report describes concurrent tuberculosis and *Nocardia* in a patient with COPD from a primary hospital in Southwest China.

## Case presentation

A 79-year-old female farmer had a medical history of COPD for more than ten years and worsening cough, sputum and gasping since January 10, 2022. She used no medication other than inhaled steroid and levofloxacin at home. On January 23, 2022, the patient was admitted to the first hospital. A computed tomography (CT) scan of the thorax showed scattered speckled nodules, patchy shadows and a few streaky shadows. Microbiological screening was not performed for unknown reasons. The main clinical diagnosis was acute exacerbation of COPD. The patient was prescribed ceftazidime (2 g, 8 hourly) for empirical treatment. Seven days after hospitalization, the patient requested to be discharged due to no remission.

**Fig. 1:**
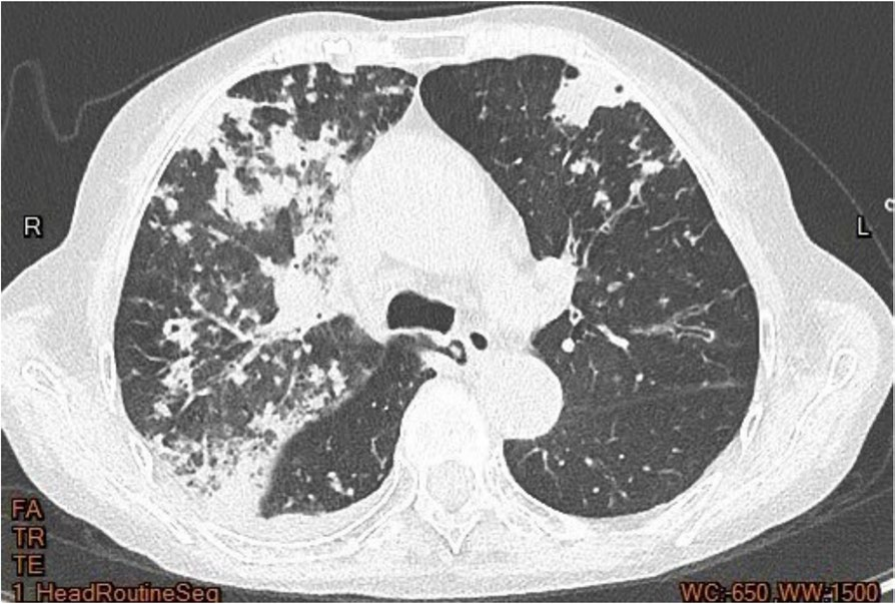
CT scan of the thorax showed scattered patchy, nodular and soft tissue masses with blurred margins in the lungs in our hospital

**Fig. 2:**
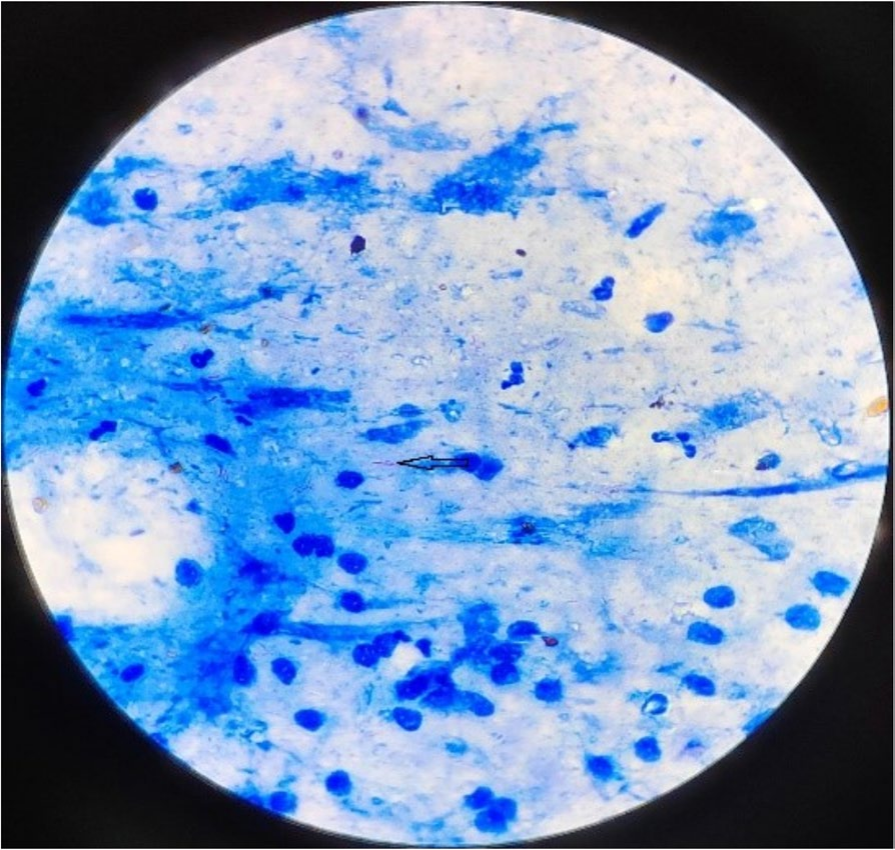
Positive acid-fast staining from sputum

**Fig. 3:**
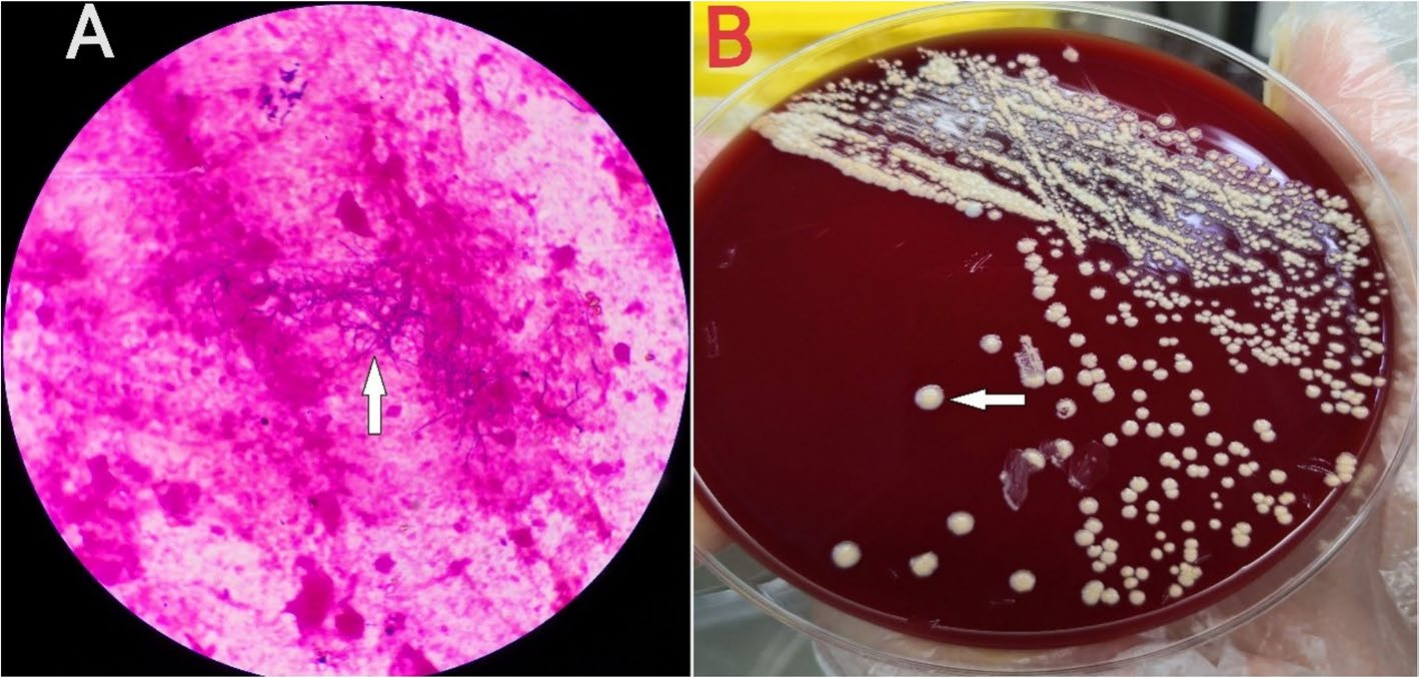
**A** Gram stain of fluid collected from BALF showed branching, filamentous gram-positive bacilli, Original magnification × 1000. **B** After 4 days of cultivation, the isolate on the blood agar identified as *N. farcinica* using MALDI-TOF–MS and 16S rRNA

On February 11, 2022, the patient was admitted to our hospital with chief complaints of recurrent cough, sputum and gasping for one month. Screening tests for respiratory 2019-novel coronavirus, influenza virus, adenovirus and respiratory syncytial virus nucleic acids in throat swab were all negative. Physical examination at admission showed body temperature 37.1 °C; heart rate 99 beats/minute, respiratory rate 21/min, BP 146/71 mmHg, and SpO_2_ 94%. A CT scan of the thorax showed scattered patchy, nodular and soft tissue masses with blurred margins in the lungs (Fig. 1). The main differential diagnosis at admission was acute exacerbation of COPD, including pulmonary bacterial infection, tuberculosis, and pulmonary fungal infection. The sputum samples were repeatedly examined. The results of acid-fast staining of sputum (Fig. 2), tuberculosis DNA, and GeneXpert were positive. Additional investigations included human immunodeficiency virus (HIV) test, 1, 3-β-D-glucan test, *Aspergillus* galactomannan, cryptococcal antigen, and antibodies for *Legion*ella and *Mycoplasma*, which were all negative, the patient's other blood indicators, such as white blood cells and the percentage of neutrophils, procalcitionin and interleukin-6 were elevated. Because the microbiological evidence was suggestive of tuberculosis, the patient was initiated on first-line anti-tuberculous therapy with isoniazid, rifampin, pyrazinamide, and ethambutol. Seven days after starting anti-tuberculous therapy, the patient developed fever and minor headache, with no remission of respiratory tract symptoms. The patient's brain CT showed patchy low density shadow in left basal ganglia, with no obvious signs of infection. After a multidisciplinary consultation to discuss the patient’s new onset of fever and minor headache, lumbar puncture and bronchoscopy were conducted to obtain cerebrospinal fluid (CSF) and bronchoalveolar lavage fluid (BALF) for a comprehensive microbiological examination. Analysis of the CSF was unremarkable, and Gram staining of BALF showed branching and beaded Gram-positive rods (Fig. 3A), which was consistent with the morphological features of *Nocardia.spp*. Acid-fast staining, tuberculosis DNA, and GeneXpert of BALF were all positive. *Nocardia.spp* was confirmed in culture four days later (Fig. 3B). Matrix-assisted laser desorption ionization-time-of-flight mass spectrometry (MALDI-TOF- MS), and sequencing of partial 16S ribosomal ribonucleic acid (RNA) were performed, which identified the *Nocardia* isolate as *N. farcinica.* The antimicrobial susceptibility pattern types in the present case were compared with those provided by CLSI standard M62 [7]. this *Nocardia* isolate was susceptible to amoxicillin/clavulanate, imipenem, amikacin, ciprofloxacin, moxifloxacin, TMP/SMX, linezolid and minocycline, resistant to ceftriaxone, tobramycin, clarithromycin, doxycycline and cefepime.

The patient was prescribed isoniazid, rifampin, pyrazinamide, and ethambutol as anti-tuberculosis treatment and TMP/SMX (2 tablets, 8 hourly) as anti-nocardiosis treatment for nineteen days. The patient showed gradual improvement, and the fever subsided. The patient insisted to be discharged due to financial reasons on March 19, 2022. The patient was intermittently followed–up telephonically and via the WeChat application. Her family members reported that the patient's condition did not worsen one month after discharge due to adherence medication, howere, we lost contact with the patient three months later.

## Discussion and conclusions

A patient with COPD may have increased susceptibility to opportunistic pathogens due to several reasons, such as changes in bronchial architecture, bacterial colonization of the lower airways, which alters ciliary motility and causes epithelial damage, the use of inhaled corticosteroid, which particularly damages the alveolar macrophages, frequent hospitalizations or antibiotic treatments, and comorbidities [5, 8]. A literature search of the PubMed database with key terms Nocardia and tuberculosis, retrieved several cases on PN that were misdiagnosed as tuberculosis in COPD patients and cases of concurrent PN and tuberculosis in HIV patients, but co-infection with *Nocardia* and *Mycobacterium tuberculosis* in COPD patients was rarely reported [9–11]. The present casea was a rare case of co-infection with *Nocardia* and *Mycobacterium tuberculosis* in a COPD patient, without systemic immunosuppressive factors.

China has a high prevalence of tuberculosis and ranks second among the 30 countries with a high burden of TB. According to the statistics of the World Health Organization (WHO), the number of new TB patients worldwide in 2020 was 9.9 million, of which Chinese TB patients accounted for 8.5% [12]. A strong correlation was found between the use of inhaled steroids and the risk of tuberculosis in COPD patients [13]. As many COPD patients require inhaled steroids therapy, they are highly susceptible to tuberculosis. Moreover, both inhaled steroids and levofloxacin are commonly used to control exacerbations of pulmonary symptoms. As a second-line drug for tuberculosis, levofloxacin may cause diagnostic pitfalls in culture media in COPD cases [14]. Meanwhile, clinical manifestations are not specific to tuberculosis, which further leads to missed diagnoses or delayed treatment. Our patient presented with acute bronchopneumonia at the previous hospital, where clinical etiological tracing was insufficient and failed to carry out etiological diagnosis. The patient’s subsequent laboratory examination at our hospital confirmed tuberculosis through sputum. However, the patient did not benefit from anti-tuberculosis treatment, and developed fever and minor headache, which prompted us to conduct a more comprehensive examination of the source and pathogens.

*Nocardia*.spp are branching, filamentous gram-positive bacilli found worldwide in soil, dust, water and decaying plant matter [15]. People are most commonly infected through inhalation of isolates in saprophytic environments. About two-thirds of all primary *Nocardia.*spp infections occur in the lungs [16]. Our patient’s initial respiratory symptoms followed by onset of fever and minor headache, inhalation was most likely the route of infection. She frequently did the farm work, which exposed her to decaying plant matter. Furthermore, COPD is frequently associated with PN and the central nervous system (CNS) is the second most common site of infection, usually through dissemination from primary pulmonary focus [4, 5]. CNS involvement can be seen in 3–26% of patients with nocardiosis [17]. However, identification of *Nocardia* is extremely difficult because nocardiosis is not well understood by clinicians and the culture of *Nocardia* is difficult. The morphological characteristics of bacteria and the progression of disease in our patient alerted us to screen for nocardiosis. Routine microscopic examination of all respiratory fluid samples is very important. The importance of microscopic examination should be highlighted because the appearance of branched unevenly colored Gram-positive bacilli is strongly suggestive of *Nocardia spp* and can guide the microbiologist to carefully search for *Nocardia* colonies. Moreover, the metagenomics platform is not available in primary hospitals. Given that the clinical signs are not specific to differentiate tuberculosis from PN, confirmed diagnosis of tuberculosis will greatly reduce other suspected pathogens. The microbiological laboratory should be notified when *Nocardia* is considered, to ensure optimal culture conditions given the potential need for longer incubation. In our patient, the diagnosis of tuberculosis was due to the standard microbiological screening procedure at our laboratory. *N. farcinica* was found in BALF owing to its unique morphological characteristics and a high degree of suspicion. Furthurmore, BALF is more reliable than sputum to determine the etiology of lower respiratory system [18]. Therefore, it is crucial to conduct a thorough microbiological evaluation since it is typically difficult to isolate and identify *Nocardia*, especially if the specimen is contaminated with other microorganisms.

The present case highlights several crucial learning points including acknowledging the possibility of co-infection with tuberculosis and PN in COPD patients. The presence of tuberculosis does not necessarily rule out other infections. A comprehensive list of differential diagnoses and meticulous diagnostic evaluation of these patients is of paramount importance to favorable clinical outcomes. Repeated microbiological examinations should be conducted in order to establish an accurate diagnosis. Therefore, microbiological and microscopy examinations of high quality sample cellection are critical in primary hospitals of China. Owing to the acuurate etiological diagnosis, the anti-infective treatement for our patient had achieved initial success. The limitation of this case report is that comprehensive follow-up of this case was not conducted, resulting in imperfect prognostic data. In the future, more data on treatment of co-infections are required.

## Data Availability

All data generated or analysed during this study are included in this published article.

## Abbreviations

COPD: Chronic obstructive pulmonary disease
CT: Computed tomography
HIV: Human immunodeficiency virus
MALDI-TOF- MS: Matrix-assisted laser desorption ionization-time-of-flight mass spectrometry
RNA: Ribosomal ribonucleic acid
TMP/SMX: Trimethoprim sulfamethoxazole
CNS: Central nervous system
BALF: Bronchoalveolar lavage fluid
CSF: Cerebrospinal fluid
WHO: World Health Organization
